# The stem region of α1,6-fucosyltransferase FUT8 is required for multimer formation but not catalytic activity

**DOI:** 10.1016/j.jbc.2022.102676

**Published:** 2022-11-03

**Authors:** Seita Tomida, Masamichi Nagae, Yasuhiko Kizuka

**Affiliations:** 1 The United Graduate School of Agricultural Science, Gifu University, Gifu, Japan; 2 Department of Molecular Immunology, Research Institute for Microbial Diseases, Osaka University, Suita, Japan; 3 Laboratory of Molecular Immunology, Immunology Frontier Research Center (IFReC), Osaka University, Suita, Japan; 4 Institute for Glyco-core Research (iGCORE), Gifu University, Gifu, Japan

**Keywords:** α1,6-fucosyltransferase, core fucose, fucosyltransferase, glycosylation, glycosyltransferase, stem region, *N*-linked glycosylation, oligomerization, BCA, bicinchoninic acid, CBB, Coomassie Brilliant Blue, cDNA, complementary DNA, CHX, cycloheximide, COPD, chronic obstructive pulmonary disease, CQ, chloroquine, ER, endoplasmic reticulum, HRP, horseradish peroxidase, IP, immunoprecipitation, OST, oligosaccharyltransferase, SAXS, small angle X-ray scattering, TBS, Tris-buffered saline

## Abstract

Alpha-1,6-fucosyltransferase (FUT8) synthesizes core fucose in *N*-glycans, which plays critical roles in various physiological processes. FUT8, as with many other glycosyltransferases, is a type-II membrane protein, and its large C-terminal catalytic domain is linked to the FUT8 stem region, which comprises two α-helices. Although the stem regions of several glycosyltransferases are involved in the regulation of Golgi localization, the functions of the FUT8 stem region have not been clarified as yet. Here, we found that the FUT8 stem region is essential for enzyme oligomerization. We expressed FUT8Δstem mutants, in which the stem region was replaced with glycine/serine linkers, in FUT8-KO HEK293 cells. Our immunoprecipitation and native-PAGE analysis showed that FUT8 WT formed a multimer but FUT8Δstem impaired multimer formation in the cells, although the mutants retained specific activity. In addition, the mutant protein had lower steady-state levels, increased endoplasmic reticulum localization, and a shorter half-life than FUT8 WT, suggesting that loss of the stem region destabilized the FUT8 protein. Furthermore, immunoprecipitation analysis of another mutant lacking a part of the stem region revealed that the first helix in the FUT8 stem region is critical for multimer formation. Our findings demonstrated that the FUT8 stem region is essential for multimer formation but not for catalytic activity, providing insights into how the FUT8 protein matures and functions in mammalian cells.

Protein glycosylation is a major posttranslational modification that is widely observed in all kingdoms of life (1). The known functions of glycosylation include the regulation of protein folding, localization, and activity, as well as cell adhesion, cell–cell interactions, and signal transduction (2). Furthermore, changes to glycan structures cause the development or improvement of various diseases, including cancer, chronic obstructive pulmonary disease (COPD), and Alzheimer’s disease (3, 4, 5), and the glycan changes are used clinically as specific disease markers (6). Therefore, elucidation of the biosynthetic mechanisms of glycan structures and how they are regulated in cells is extremely important for a detailed understanding of the physiological and pathological functions of glycans and provides insights into ways to control these functions.

Glycans on proteins are classified into *N*-glycan, *O*-glycan, *C*-mannose, and glycosylphosphatidylinositol according to their core structures (1, 7). Among them, asparagine-linked *N*-glycans, the main focus of this study, are synthesized stepwise in the protein secretory pathway (8). In the endoplasmic reticulum (ER), the common precursor *N*-glycan, Glc_3_Man_9_GlcNAc_2_, is transferred to an asparagine residue in the consensus sequence (Asn-X-Ser/Thr) by oligosaccharyltransferase (OST) (9). While the *N*-glycosylated protein is transported from the ER to the Golgi, the *N*-glycan undergoes stepwise modifications by glycosidases and glycosyltransferases. As a result, various *N*-glycans with complex structures are synthesized in a number of cell type–specific, protein-specific, glycosylation site–specific, and disease-specific manners. To date, approximately 180 glycosyltransferases have been identified in humans, and their expression levels, subcellular localization, and activities affect the expression of the specific glycans. However, the regulatory mechanisms for the expression, activity, and intracellular transport of glycosyltransferases have not been fully elucidated.

In *N*-glycans, fucose residue can be attached to either core or termini. The fucose residue, which is connected *via* an α1,6-linkage to the reducing end GlcNAc of *N*-glycans, is called core fucose (Fig. 1*A*) (10, 11). Alpha-1,6-fucosyltransferase (FUT8) is the sole enzyme responsible for the synthesis of core fucose (11), and FUT8 transfers fucose from GDP-fucose to the GlcNAc residue in the Golgi (Fig. 1*A*). Core fucose is involved in various biological processes, such as epidermal growth factor receptor, T-cell receptor, and Toll-like receptor signaling, and in various diseases, including COPD, melanoma, lung cancer, and the exacerbation of COVID-19 (4, 12, 13, 14, 15, 16, 17). In addition, removing core fucose from IgG_1_ *N*-glycan causes a dramatic rise in antibody-dependent cellular cytotoxicity (18), which has already been clinically used to functionally modify antibody drugs. These findings indicate that core fucose plays important physiological and pathological roles and is a drug target for cancer and COPD. In contrast, regulatory mechanisms for core fucose biosynthesis are largely unknown.

**Figure 1:**
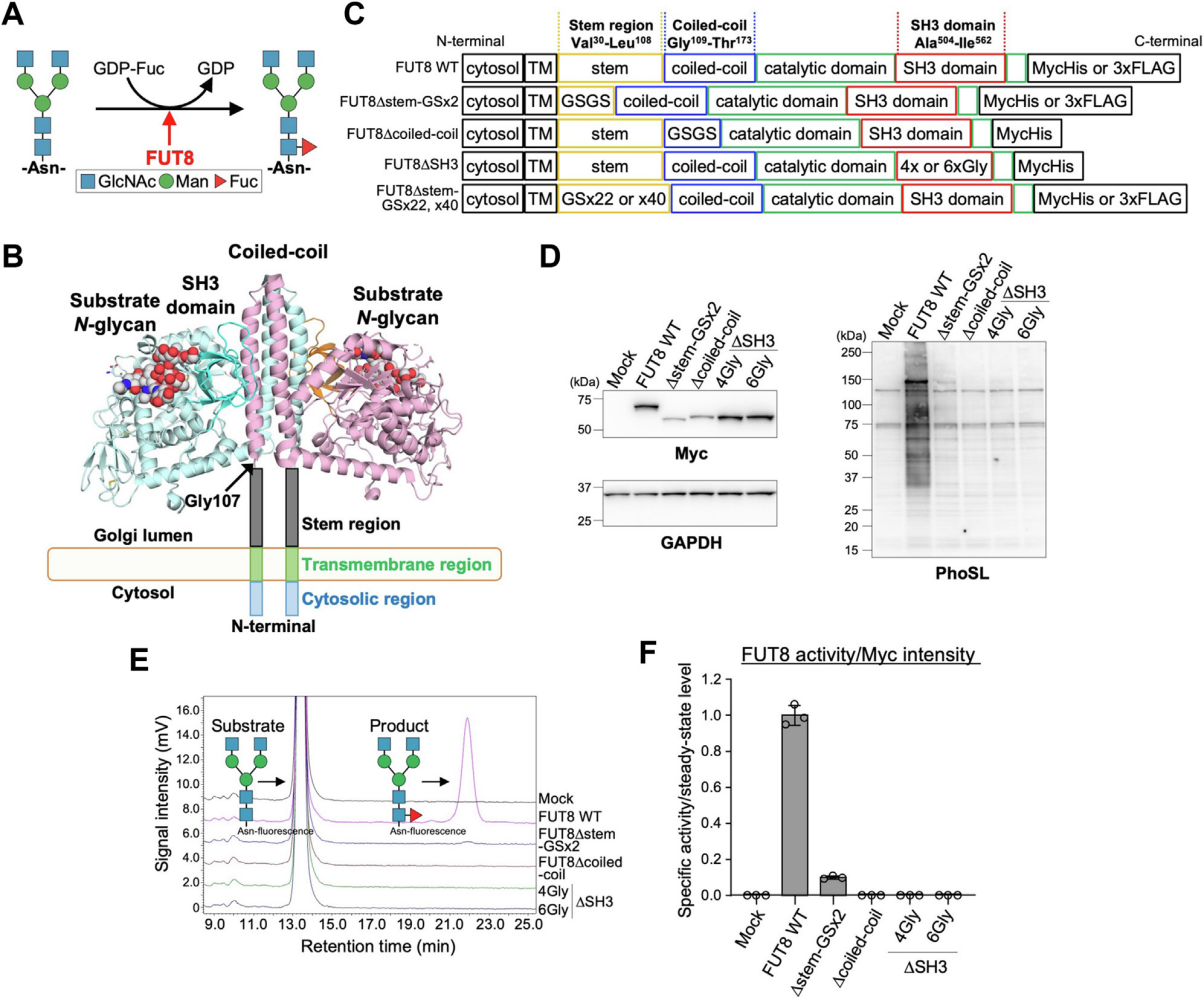
**Activity of FUT8Δstem-GSx2.***A*, schematic diagram of core fucosylation by FUT8. *B*, structure of FUT8 dimer (PDB code: 6VLD). Space-filled models are shown with substrate *N*-glycan. FUT8 stem region consists of Val30 to Leu108. *C*, schematic structure of FUT8 WT and mutants used in this study. *D*, HEK293 FUT8KO cells expressing FUT8 WT or mutants were analyzed by Western blotting with anti-Myc or anti-GAPDH antibody and by lectin blotting with biotinylated core fucose-specific PhoSL (expected molecular weight: FUT8 WT, 72 kDa; FUT8Δstem-GSx2, 62 kDa; FUT8Δcoiled-coil, 64 kDa; FUT8ΔSH3-4Gly, 65 kDa; FUT8ΔSH3-6Gly, 65 kDa). *E*, lysates of HEK293 FUT8KO cells expressing FUT8 WT or mutants were incubated with an acceptor substrate (GnGn-bi-Asn-PNSNB) and GDP-fucose, and the reaction mixture was analyzed by reverse-phase HPLC. *F*, FUT8 activity relative to that of FUT8 WT calculated from peak area in (*E*) (*n* = 3, means ± SD).

FUT8 is a type II transmembrane protein consisting of an N-terminal cytoplasmic region, transmembrane region, stem region (Val30-Leu108), coiled-coil domain (Gly109-Thr173), catalytic domain (Asp174-Asn503 and Glu563-Lys575), and Src homology 3 (SH3) domain (Ala504-Ile562) (Fig. 1*B*) (19, 20). The functions of all domains and regions, other than the catalytic domain, were unclear for a long time, but recent studies have gradually uncovered the importance of these noncatalytic domains. We recently reported that the FUT8 SH3 domain is essential for the enzymatic activity and partial cell surface localization of FUT8 and that FUT8 binds with ribophorin I (RPN1), a subunit of OST that interacts with ribosomes to facilitate *N*-glycosylation by OST in the ER, in a manner dependent on the FUT8 SH3 domain (21). In addition, knockdown of RPN1 and glycomic analysis showed that RPN1 positively regulates FUT8 activity and core fucosylation in cells. Another research group showed that the coiled-coil domain is involved in the enzymatic activity and homophilic interaction of FUT8 (22). In contrast to SH3 and the coiled-coil domains, there have been no reports on the 3D structure and functions of the FUT8 stem region. The stem region is commonly found in many glycosyltransferases and regulates Golgi localization or homophilic interactions with glycosyltransferases (23). In addition, in the case of protein *O*-mannose β-1,2-*N*-acetylglucosaminyltransferase (POMGnT1), the protein responsible for muscular dystrophy, the stem region functions as a lectin domain and binds to core M3 glycan to improve *O*-mannosylation (24). Based on these reports, we reasoned that the FUT8 stem region also has some functions in core fucose biosynthesis, such as the regulation of intracellular transport or homophilic interactions of FUT8.

In this study, to understand the functions of the FUT8 stem region, we investigated the properties of FUT8 mutants lacking the stem region and clarified that this region is required for multimer formation and the stabilization of FUT8. These findings provide new insights into how the expression and activity of FUT8 are regulated in cells.

## Results

### Specific sequence of FUT8 stem region is dispensable for FUT8 enzymatic activity

To investigate the functions of the FUT8 stem region, we constructed the expression plasmids encoding a FUT8 mutant, FUT8Δstem-GSx2, in which the stem region was replaced with a glycine and serine repeat (GSGS) (Fig. 1*C*). As controls, we also prepared plasmids for other FUT8 deletion mutants, in which the coiled-coil (FUT8Δcoiled-coil) or SH3 domain (FUT8ΔSH3) was deleted. To examine the steady-state levels and activities of these mutants in living cells, HEK293 FUT8KO (25) cells were transfected with expression plasmids encoding Myc-tagged FUT8 WT, FUT8Δstem-GSx2, FUT8Δcoiled-coil, or FUT8ΔSH3, and the cellular proteins were analyzed by Western and lectin blotting (Fig. 1*D*). Western blotting with an anti-Myc tag antibody showed that the steady-state levels of FUT8Δstem-GSx2 and FUT8Δcoiled-coil were significantly lower than those of FUT8 WT and FUT8ΔSH3 (26% and 41% of the steady-state levels of FUT8 WT for FUT8Δstem-GSx2 and FUT8Δcoiled-coil, respectively) (Fig. 1*D*, left upper). Core fucose was detected using the specific binding of PhoSL lectin (Fig. 1*D*, right) (26). PhoSL signals in the FUT8 WT sample were dramatically increased compared with those in the mock-transfected sample, confirming that PhoSL specifically recognizes the core fucose synthesized by FUT8 and that the biosynthetic activity of FUT8 in cells can be evaluated by PhoSL signals. In contrast to FUT8 WT, PhoSL signals in FUT8Δstem-GSx2-expressing cells were very low and those of FUT8Δcoiled-coil were almost undetectable (Fig. 1*D*, right), indicating that these mutants were almost unfunctional in the cells. The catalytically dead FUT8ΔSH3 also generated no PhoSL signals, consistent with our previous report (21). To examine whether the enzymatic activities of FUT8Δstem-GSx2 and FUT8Δcoiled-coil were also absent, we next measured the *in vitro* activities of FUT8 WT and its mutants using an established HPLC-based method (Fig. 1, *E* and *F*) (27, 28). The lysates of FUT8KO cells expressing FUT8 WT or its mutants were incubated with the fluorescently labeled acceptor substrate GnGnbiAsn-PNS and the donor substrate GDP-fucose, and the reaction mixtures were analyzed by HPLC to separate and quantify the acceptor substrate and its core fucosylated product. The enzymatic activities of FUT8 and its mutants calculated from HPLC analysis were normalized to the steady-state levels of the enzymes (anti-Myc intensity in Fig. 1*D*). FUT8Δstem-GSx2 showed very much lower enzymatic activity than FUT8 WT, and no activity of FUT8Δcoiled-coil was detected (Fig. 1*F*), and the latter finding is consistent with a previous report in which a soluble FUT8 mutant lacking the N terminus to coiled-coil domain was inactive (22). In contrast, previous reports showed that soluble FUT8 mutants partially or fully lacking the stem region were all active (22, 29, 30), suggesting that the FUT8 stem region is not essential for the enzyme activity itself and that FUT8Δstem-GSx2 became unfunctional in cells for another reason.

**Figure 2:**
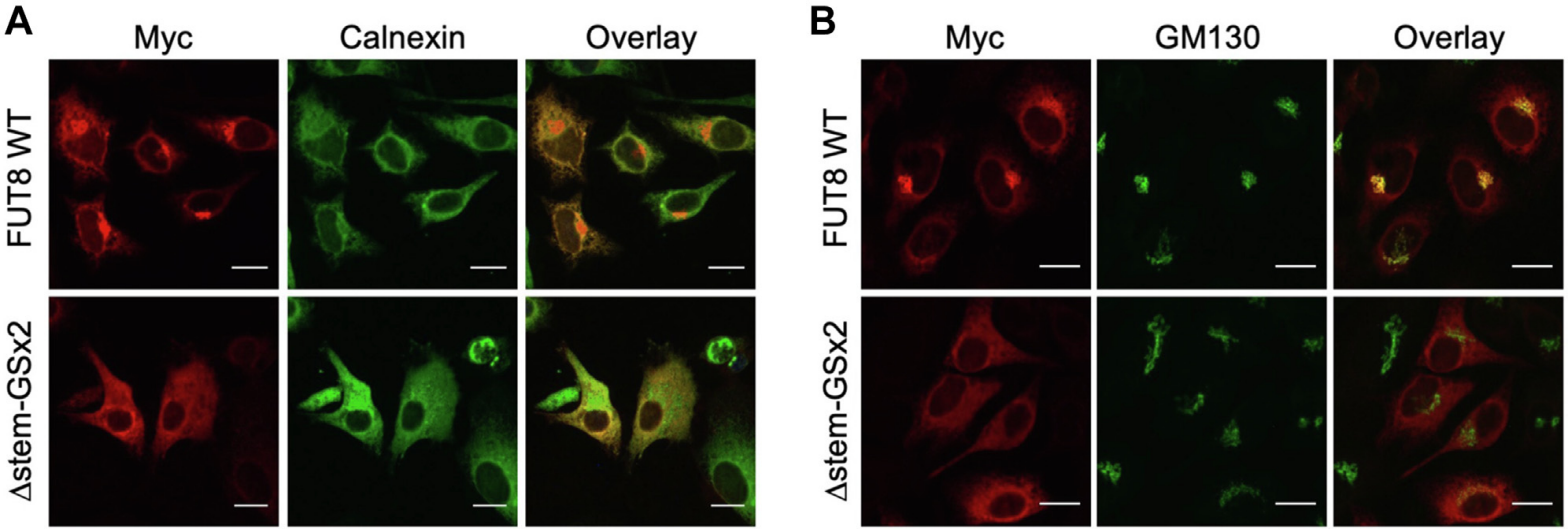
**Localization of FUT8Δstem-GSx2.***A*, immunofluorescence staining of HeLa cells expressing FUT8 WT and Δstem-GSx2, respectively (*Red*: Myc, *Green*: calnexin, scale bar: 20 μm). *B*, immunofluorescence staining of HeLa cells expressing FUT8 WT and Δstem-GSx2, respectively (*Red*: Myc, *Green*: GM130, scale bar: 20 μm).

Because the GSx2 linker (4 amino acids) in the FUT8Δstem-GSx2 mutant is dramatically shorter than the native FUT8 stem region (79 amino acids), we hypothesized that FUT8Δstem-GSx2 was misfolded due to the shortness of the linker, resulting in its accumulation in the ER. To verify this possibility, the localization of FUT8 WT and FUT8Δstem-GSx2 was investigated by immunofluorescence staining (Fig. 2, *A* and *B*). We used HeLa cells for the immunofluorescence staining because they more strongly adhere to the chamber slides and are flatter in shape than HEK293 cells, which allowed us to observe the localization of FUT8 more clearly. FUT8 WT mostly overlapped with the Golgi marker GM130 but barely with the ER marker calnexin. In contrast, FUT8Δstem-GSx2 well overlapped with calnexin, suggesting that FUT8Δstem-GSx2 was misfolded and entrapped in the ER.

**Figure 3:**
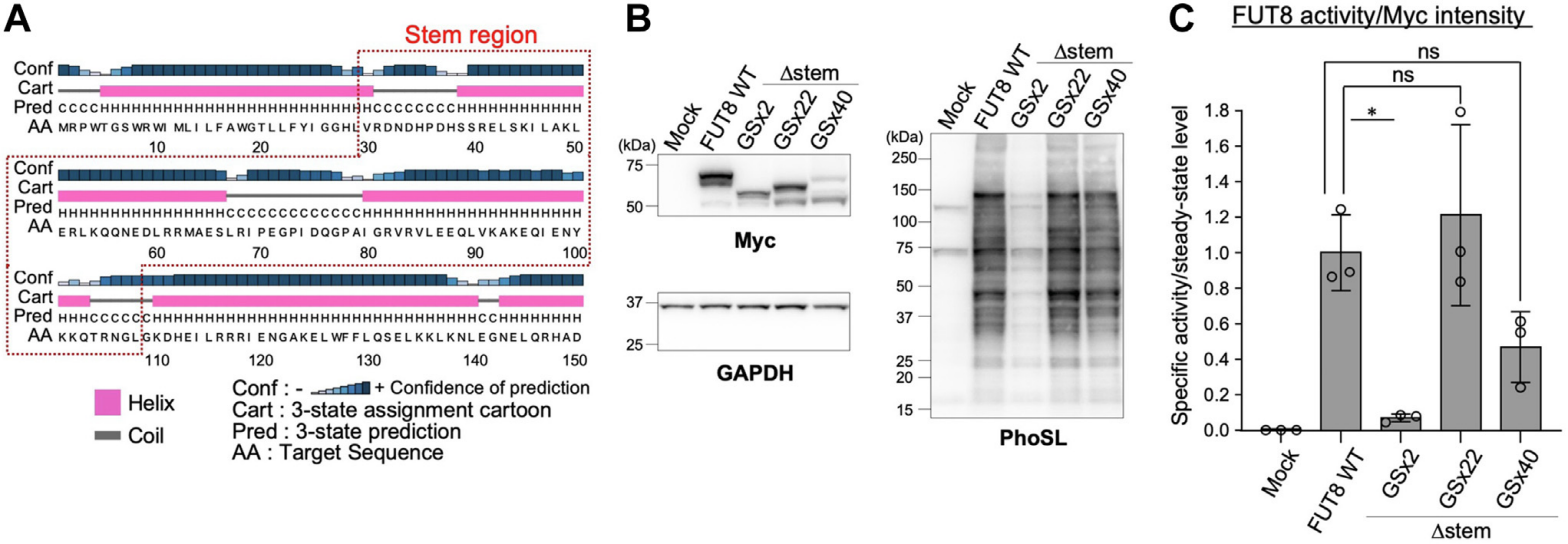
**Enzymatic activity of FUT8Δstem-GSx22 and FUT8Δstem-GSx40.***A*, prediction of secondary structure of FUT8 stem region using PSIPRED 4.0. *B*, HEK293 FUT8KO cells expressing FUT8 WT or Δstem mutants were analyzed by Western blotting with anti-Myc or anti-GAPDH antibody and by lectin blotting with PhoSL (expected molecular weight: FUT8Δstem-GSx22, 65 kDa; FUT8Δstem-GSx40, 68 kDa). *C*, enzymatic activity of cell lysates expressing FUT8 WT or Δstem mutants was measured using the same method as in (Fig. 1*E*). The activity was divided by the intensity of anti-Myc signals in (*B*). The intensity of both upper and lower bands in the anti-Myc blots were included for quantification because the lower band (approximately 50 kDa) is reactive with anti-Myc antibody and likely contains all part of the C-terminal catalytic region. Graph shows enzyme activity relative to that of FUT8 WT (*n* = 3, means ± SD, ns, not significant, ∗*p* < 0.05, Tukey’s multiple comparison test).

To investigate whether the FUT8 stem region forms a linear or globular tertiary structure, we predicted the secondary structure of the FUT8 stem region (Fig. 3*A*). Predictions showed that it consists of two helices, suggesting that it takes a long linear tertiary structure. Therefore, it is possible that FUT8 requires a structurally long stem region for its folding, and the GSx2 linker is too short to express active FUT8 in cells. We thus constructed two additional expression plasmids encoding Myc-tagged FUT8Δstem-GSx40 and FUT8Δstem-GSx22, which have 80 and 44 amino acids (comprising repeats of glycine-serine, which do not form a specific secondary structure), respectively, instead of the FUT8 stem region (Fig. 1*C*). These mutants were expressed in HEK293 FUT8KO cells, and the cellular proteins were analyzed by Western and PhoSL blotting (Fig. 3*B*). Anti-Myc blotting showed that FUT8Δstem-GSx22 and -GSx40 formed two bands. The levels of the upper bands of FUT8Δstem-GSx22 and FUT8Δstem-GSx40 were significantly lower than that of FUT8 WT and instead the levels of the lower bands (∼50 kDa) of FUT8Δstem-GSx22 and FUT8Δstem-GSx40 were increased compared with FUT8 WT (Fig. 3*B*, left). The two bands were not due to *N*-glycoforms because there is no *N*-glycosylation site in FUT8, implying that these Δstem mutants were somewhat unstable in the cells. The strength of the PhoSL signals in the FUT8Δstem-GSx22 sample was similar to that of FUT8 WT (Fig. 3*B*, right), and the signal in the FUT8Δstem-GSx40 sample was slightly decreased compared with that in the FUT8 WT samples but very much higher than that in FUT8Δstem-GSx2. The *in vitro* enzymatic activities of FUT8Δstem-GSx22 and FUT8Δstem-GSx40, which were measured by the HPLC-based method and normalized to their steady-state levels, were markedly higher than that of FUT8Δstem-GSx2 (Fig. 3*C*, FUT8Δstem-GSx2, 0.0695; FUT8Δstem-GSx22, 1.21; FUT8Δstem-GSx40, 0.469). Together, these findings suggest that the specific native sequence of the stem region is not required for the FUT8 enzyme activity and that FUT8Δstem-GSx22 and FUT8Δstem-GSx40 are more suitable than FUT8Δstem-GSx2 for evaluating the functions of the FUT8 stem region.

### FUT8 mutants with non-native stem regions lead to inefficient trafficking and proteolysis

**Figure 4:**
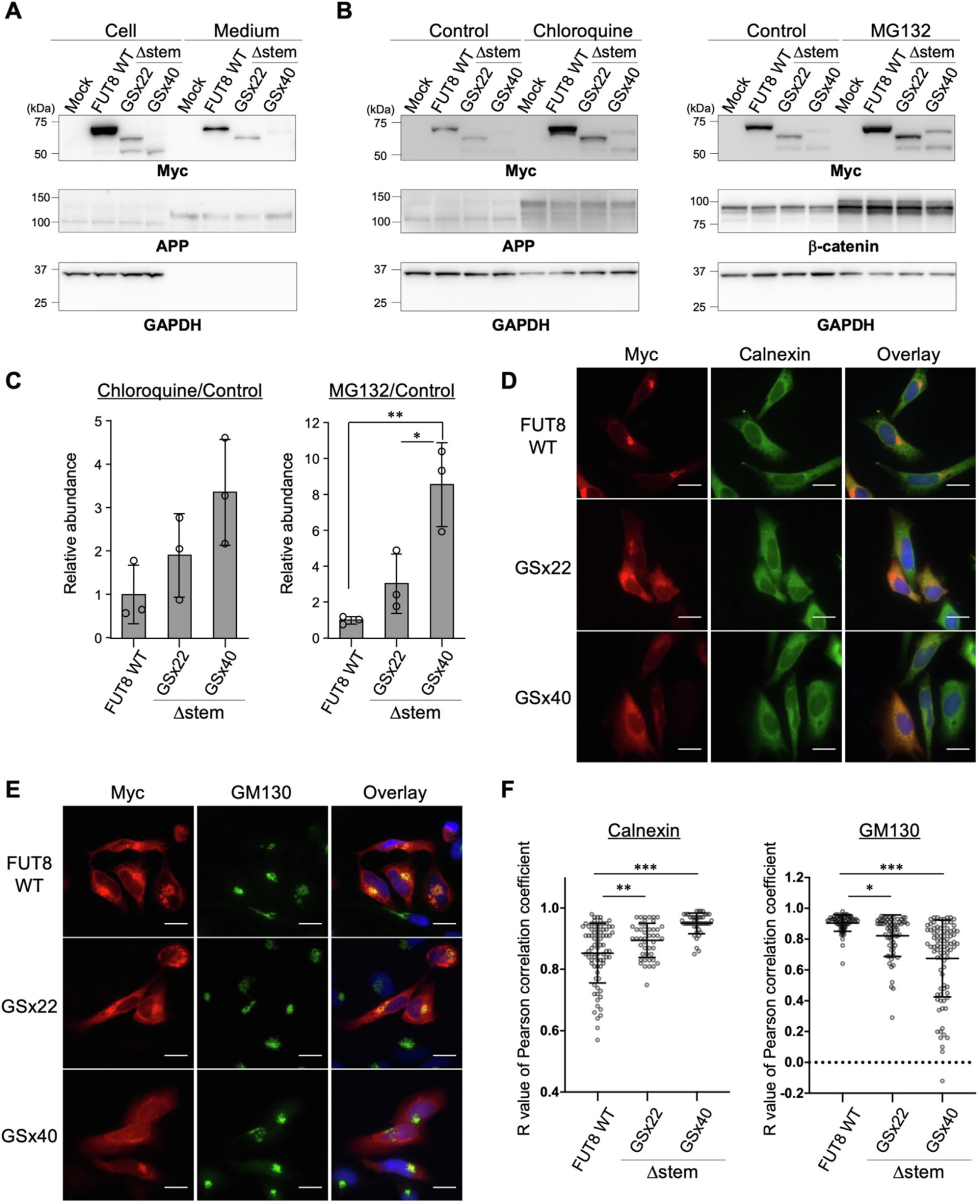
**Enhanced degradation of FUT8Δstem-GSx22 and FUT8Δstem-GSx40.***A*, proteins in cells and culture media from HEK293 FUT8KO cells expressing FUT8 WT or Δstem mutants were analyzed by Western blotting with anti-Myc, anti-APP, or anti-GAPDH antibody. Secreted proteins in the media were precipitated with ethanol (expected molecular weight: APP, 110, 120, and 130 kDa). *B*, HEK293 FUT8KO cells expressing FUT8 WT or Δstem mutants were treated with DMSO (control), chloroquine, or MG132 for 48 h. Cell lysates were analyzed by Western blotting with anti-Myc, anti-APP, anti-GAPDH, or anti-β-catenin antibody (expected molecular weight: β-catenin, 92 kDa). *C*, quantification of the intensity of anti-Myc signal in (*B*). The intensity of the sample treated with chloroquine or MG132 relative to that of control was calculated. Graph shows calculated intensity relative to that of FUT8 WT (*n* = 3, means ± SD, ∗*p* < 0.05, ∗∗*p* < 0.01, Tukey’s multiple comparison test). *D*, immunofluorescence staining of HeLa cells expressing FUT8 WT, Δstem-GSx22, or Δstem-GSx40 (*Red*: Myc, *Green*: calnexin, *Blue*: DAPI, scale bar: 20 μm). *E*, immunofluorescence staining of HeLa cells expressing FUT8 WT, Δstem-GSx22, or Δstem-GSx40 (*Red*: Myc, *Green*: GM130, *Blue*: DAPI, scale bar: 20 μm). *F*, the R values of Pearson correlation coefficients between the calnexin or GM130 and Myc signal in each cell were calculated from images of immunofluorescence staining by Coloc 2 (means ± SD, ∗*p* < 0.05, ∗∗*p* < 0.01, ∗∗∗*p* < 0.001, Tukey’s multiple comparison test). DAPI, 4′,6-diamidino-2-phenylindole; DMSO, dimethyl sulfoxide.

As shown in Figure 3*B*, the total steady-state levels of FUT8Δstem-GSx22 and FUT8Δstem-GSx40 were both decreased compared with FUT8 WT. As possible mechanisms for this decreased expression of the mutants, we assumed the extracellular secretion or intracellular degradation of FUT8Δstem-GSx22 and FUT8Δstem-GSx40 was promoted. As FUT8 activity was detected in human serum (31), FUT8 is natively secreted from cells, like many other glycosyltransferases (32). Therefore, we first investigated whether secretion of the FUT8Δstem mutants is promoted. To examine the level of secreted FUT8, proteins in the culture media of HEK293 FUT8KO cells expressing Myc-tagged FUT8 WT, FUT8Δstem-GSx22, or FUT8Δstem-GSx40 were prepared by ethanol precipitation and analyzed by Western blotting (Fig. 4*A*). Amyloid precursor protein (APP) was also investigated as a control, as it is known to be secreted into culture media (33). In the case of FUT8 WT, the anti-Myc signal was detected in the media, indicating that FUT8 WT was secreted out of the cells. The amounts of FUT8Δstem-GSx22 and FUT8Δstem-GSx40 in the media were lower than that of FUT8 WT, which was similar to the cell samples. This suggests that the extracellular secretion of FUT8Δstem-GSx22 and FUT8Δstem-GSx40 is comparable to that of FUT8 WT and that the decreased steady-state levels of FUT8Δstem-GSx22 and FUT8Δstem-GSx40 in cells were not caused by a facilitation of extracellular secretion.

Next, we focused on the intracellular degradation of FUT8Δstem-GSx22 and FUT8Δstem-GSx40. To examine proteasomal and lysosomal degradation, HEK293 FUT8KO cells expressing FUT8 WT, FUT8Δstem-GSx22, or FUT8Δstem-GSx40 were treated with a lysosome inhibitor, chloroquine (CQ), or a proteasome inhibitor, MG132 for 48 h, and the accumulation of the proteins in the cells was investigated by Western blotting (Fig. 4, *B* and *C*). Accumulation of APP following CQ treatment (Fig. 4*B*, left) and of β-catenin following MG132 treatment (Fig. 4*B*, right) confirmed that lysosome and proteasome functions were inhibited by these compounds, as expected (34, 35). The levels of FUT8 WT, FUT8Δstem-GSx22, and FUT8Δstem-GSx40 were all increased by CQ treatment (Fig. 4, *B*, left and *C*, left, FUT8Δstem-GSx22 and FUT8Δstem-GSx40 were 1.90 and 3.36, respectively). In contrast, following MG132 treatment, the abundance of FUT8 WT was only slightly increased, while the signals for FUT8Δstem-GSx22 and FUT8Δstem-GSx40 were more clearly increased (Fig. 4*B*, right and 4C, right, FUT8Δstem-GSx22 and FUT8Δstem-GSx40 were 3.03 and 8.56, respectively). These findings demonstrated that FUT8 WT is basically degraded in lysosomes and that the aberrant proteasomal degradation of FUT8Δstem-GSx22 and FUT8Δstem-GSx40 was facilitated, which probably caused a decrease in the steady-state levels of FUT8Δstem-GSx22 and FUT8Δstem-GSx40. To further corroborate these findings, the subcellular localizations of FUT8Δstem-GSx22 and FUT8Δstem-GSx40 were investigated by immunofluorescence staining (Fig. 4, *D* and *E*). Almost all FUT8 WT were localized to the Golgi, as also shown by the data in Figure 2*B*. In contrast, FUT8Δstem-GSx22 was localized to both the Golgi and ER, and FUT8Δstem-GSx40 was mainly localized to the ER. The R values of Pearson correlation coefficients between the calnexin or GM130 and the Myc signals confirmed the increased colocalization of FUT8Δstem-GSx40 with calnexin and the decreased colocalization of FUT8Δstem-GSx40 with GM130 (Fig. 4*F*). These data showed that FUT8Δstem-GSx22 and FUT8Δstem-GSx40 were more localized to the ER than FUT8 WT, suggesting that FUT8 mutant proteins lacking the stem region are partially misfolded in the ER for some reasons and subsequently degraded in proteasomes.

**Figure 5:**
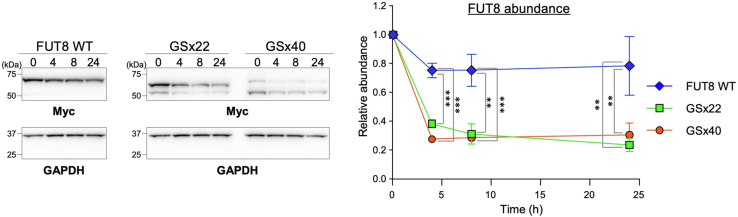
**Degradation rate of FUT8Δstem-GSx22 and FUT8Δstem-GSx40.** HEK293 FUT8KO cells expressing FUT8 WT, Δstem-GSx22, or Δstem-GSx40 were treated with cycloheximide for 0, 4, 8, or 24 h, and the cell lysates were analyzed by Western blotting with anti-Myc or anti-GAPDH antibody. Graph shows the abundance (calculated from the intensity of anti-Myc blotting) of FUT8 WT, Δstem-GSx22, and Δstem-GSx40, relative to that of the respective 0 h sample (*n* = 3, means ± SD, ∗∗*p* < 0.01, ∗∗∗*p* < 0.001, Tukey’s multiple comparison test).

To assess the degradation rates of FUT8Δstem-GSx22 and FUT8Δstem-GSx40, we next carried out chase experiments using a translation inhibitor cycloheximide (CHX). After adding CHX to cells expressing FUT8 WT or mutants, the cells were chased for different periods to monitor the degradation of FUT8 proteins by Western blotting (Fig. 5). After 24 h chase, the abundance of FUT8 WT was decreased to about 70% of the initial amount. However, FUT8Δstem-GSx22 and FUT8Δstem-GSx40 were decreased to about 30% to 40% of the initial amounts, even after 4 h chase (Fig. 5), demonstrating that the degradation rates of FUT8Δstem-GSx22 and FUT8Δstem-GSx40 were significantly faster than that of FUT8 WT. These results clarified that interference with the FUT8 stem structure causes destabilization of the FUT8 protein.

### Changes in the FUT8 stem region alter the formation of higher order complexes

To understand why FUT8Δstem-GSx22 and FUT8Δstem-GSx40 are degraded faster than the WT protein, we focused on the formations of multimers (or higher order complexes) of these mutants, as it was recently reported that FUT8 forms a functional dimer (29, 30). For instance, purified soluble FUT8 forms a dimer both in crystals in a complex with substrates and in solution, the latter of which was shown by small angle X-ray scattering (SAXS) (30). Molecular dynamics simulation also showed that dimer formation of FUT8 is important for maintaining the structure of the catalytic domain (30). Moreover, the stem region of silkworm FUT8 contains a cysteine residue (not conserved in humans) that forms a disulfide bond for dimerization (36). Based on these reports, we hypothesized that the stem region of human FUT8 is also required for dimerization and that the misfolding and degradation of FUT8Δstem-GSx22 and FUT8Δstem-GSx40 are the results of a failure in dimer formation.

**Figure 6:**
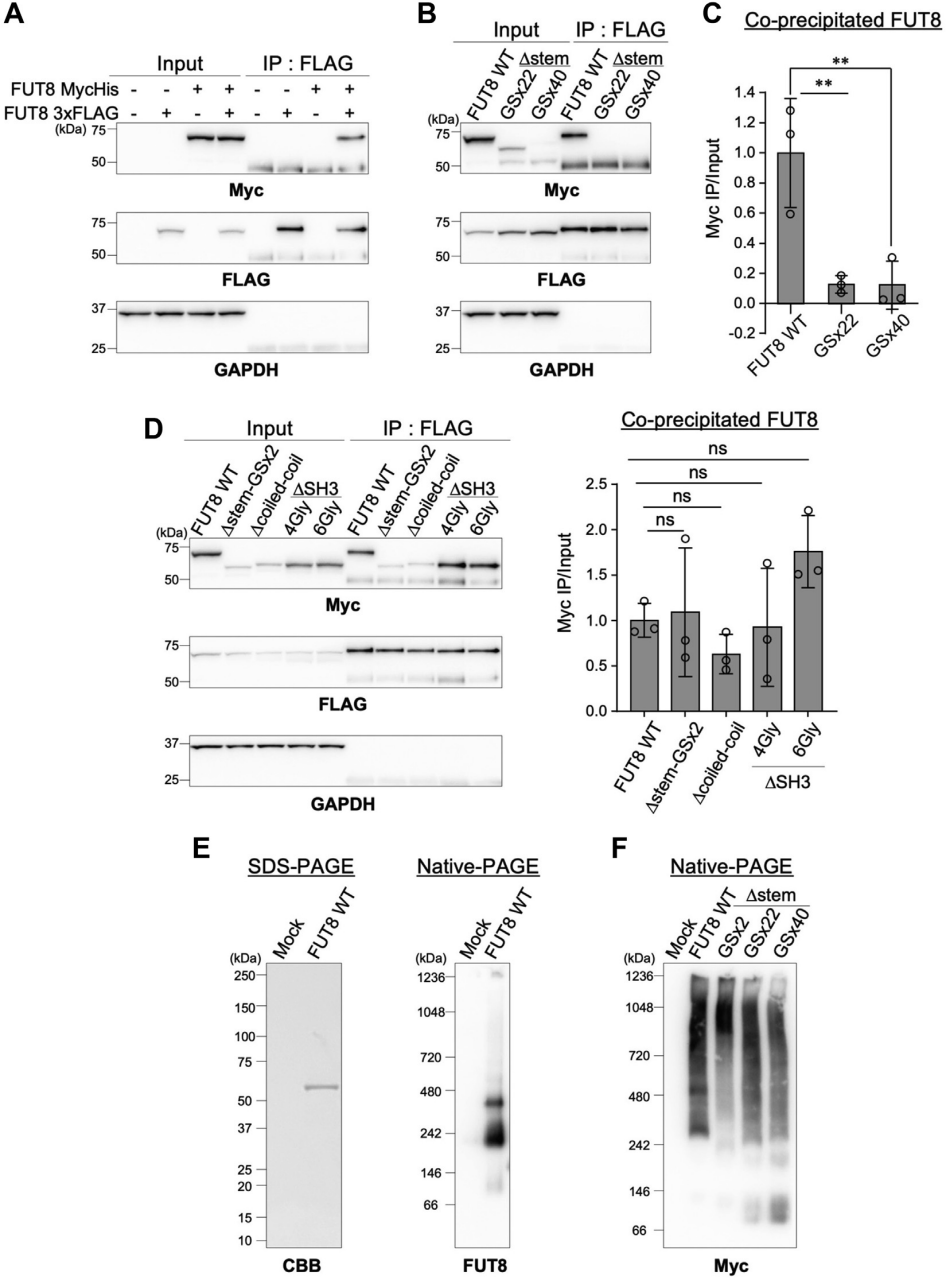
**Impaired multimer formation of FUT8Δstem-GSx22 and FUT8Δstem-GSx40.***A*, lysates of HEK293 FUT8KO cells expressing Myc-FUT8, 3xFLAG-FUT8, or both were incubated with anti-FLAG antibody-conjugated magnetic beads to precipitate 3xFLAG-FUT8. Proteins in the lysates (input) or bound to the beads (IP: FLAG) were analyzed by Western blotting with anti-Myc, anti-FLAG, or anti-GAPDH antibody. *B*, lysates of HEK293 FUT8KO cells coexpressing Myc-FUT8 WT, Δstem-GSx22, or Δstem-GSx40 with 3xFLAG-FUT8 WT were subjected to IP with anti-FLAG antibody. Proteins in the lysates (input) or bound to the beads (IP: FLAG) were analyzed by Western blotting with anti-Myc, anti-FLAG, or anti-GAPDH antibody. *C*, quantification of Myc-FUT8 WT, Δstem-GSx22, or Δstem-GSx40 coprecipitated with 3xFLAG-FUT8. The intensity of Myc-FUT8 or its mutants coprecipitated with 3xFLAG-FUT8 was divided by that of the respective input sample in the anti-Myc blot shown in (*B*). The graph shows the signals relative to that of FUT8 WT (*n* = 3, means ± SD, ∗∗*p* < 0.01, Tukey’s multiple comparison test). *D*, lysates of HEK293 FUT8KO cells coexpressing Myc-FUT8 WT, Δstem-GSx2, Δcoiled-coil, or ΔSH3 with 3xFLAG-FUT8 were subjected to IP with anti-FLAG antibody. Proteins in lysates (input) or bound to the beads (IP: FLAG) were analyzed by Western blotting with anti-Myc, anti-FLAG, or anti-GAPDH antibody. The graph shows quantification of the coprecipitated FUT8 or its mutant signals, which were calculated same as (*C*) (*n* = 3, means ± SD, ns, not significant, Tukey’s multiple comparison test). *E*, soluble His-FUT8 was purified from COS7 culture medium using a Ni^2+^ column. Purified FUT8 was separated by SDS-PAGE, followed by CBB staining (*left*) or by native-PAGE, followed by Western blotting with anti-FUT8 antibody (*right*). *F*, proteins in the lysates of HEK293 FUT8KO cells expressing FUT8 WT or mutants were separated by native-PAGE, followed by Western blotting with anti-Myc antibody. CBB, Coomassie Brilliant Blue; IP, immunoprecipitation.

To test this hypothesis, we first attempted to detect the intracellular multimer (hereafter “multimer” also includes other higher order complexes) of FUT8 by immunoprecipitation (IP). We expressed Myc-tagged FUT8 and 3xFLAG-tagged FUT8 in HEK293 FUT8KO cells, immunoprecipitated FLAG-tagged FUT8, and examined whether Myc-tagged FUT8 was coprecipitated (Fig. 6*A*). When FLAG-tagged FUT8 only or Myc-tagged FUT8 only was expressed, the Myc-FUT8 signal was not detected in the immunoprecipitate. Whereas, when both Myc-tagged and FLAG-tagged FUT8 were coexpressed, a Myc-FUT8 signal was clearly detectable (Fig. 6*A*, eighth lane). This suggests that the FUT8 WT interacted with another FUT8 molecule to form a multimer in the cells and that the multimer can be detected by IP-Western experiments using different tags. Using this IP-Western system, the multimer formation between Myc-tagged FUT8Δstem-GSx22 or FUT8Δstem-GSx40 and FLAG-tagged FUT8 WT was investigated. Coprecipitation of Myc-tagged FUT8-WT, FUT8Δstem-GSx22, or FUT8Δstem-GSx40 with FLAG-tagged FUT8 was evaluated by dividing the intensity of the anti-Myc signals of the IP samples by those of respective input samples. We observed that Myc-tagged FUT8Δstem-GSx22 and FUT8Δstem-GSx40 barely coprecipitated with FLAG-tagged FUT8 WT (Fig. 6*B*, fifth and sixth lanes, and Fig. 6*C*, 0.127 and 0.122 for FUT8Δstem-GSx22 and FUT8Δstem-GSx40, respectively). In contrast, FUT8Δcoiled-coil and FUT8ΔSH3 interact with FUT8 WT to form a multimer (Fig. 6*D*). Collectively, these results indicate that the FUT8 stem region is essential for multimer formation in cells.

The multimer formation of FUT8 in cells was further investigated by native-PAGE, which allowed us to analyze protein complexes without them being denatured. First, we investigated whether the FUT8 multimer could be detected by native-PAGE using purified soluble FUT8, which consisted of Arg68-Lys575 and was purified using a Ni^2+^ column (Fig. 6*E*). Coomassie Brilliant Blue (CBB) staining after SDS-PAGE showed a single band of purified FUT8 between 50 kDa and 75 kDa (Fig. 6*E*, left). When native-PAGE was performed using the same enzyme solution, several bands over 146 kDa were detected, which indicated that soluble FUT8 formed multimers in solution, consistent with the previous SAXS analysis (30), and that native-PAGE can detect FUT8 multimers. We then analyzed the multimer formation of FUT8 WT, FUT8Δstem-GSx22, and FUT8Δstem-GSx40 expressed in HEK293 FUT8KO cells (Fig. 6*F*). In the FUT8 WT sample, several large FUT8 complexes were observed. In contrast, the amounts of FUT8Δstem-GSx22 and FUT8Δstem-GSx40 complexes between 242 kDa and 480 kDa were decreased, and the amounts of the smaller molecular species between 66 kDa and 146 kDa were increased. These results demonstrated that deletion of the FUT8 stem region caused a failure in multimer formation, which may have resulted in the instability and faster degradation of FUT8Δstem-GSx22 and FUT8Δstem-GSx40.

### Helix 1 in FUT8 stem region is essential for multimer formation

**Figure 7:**
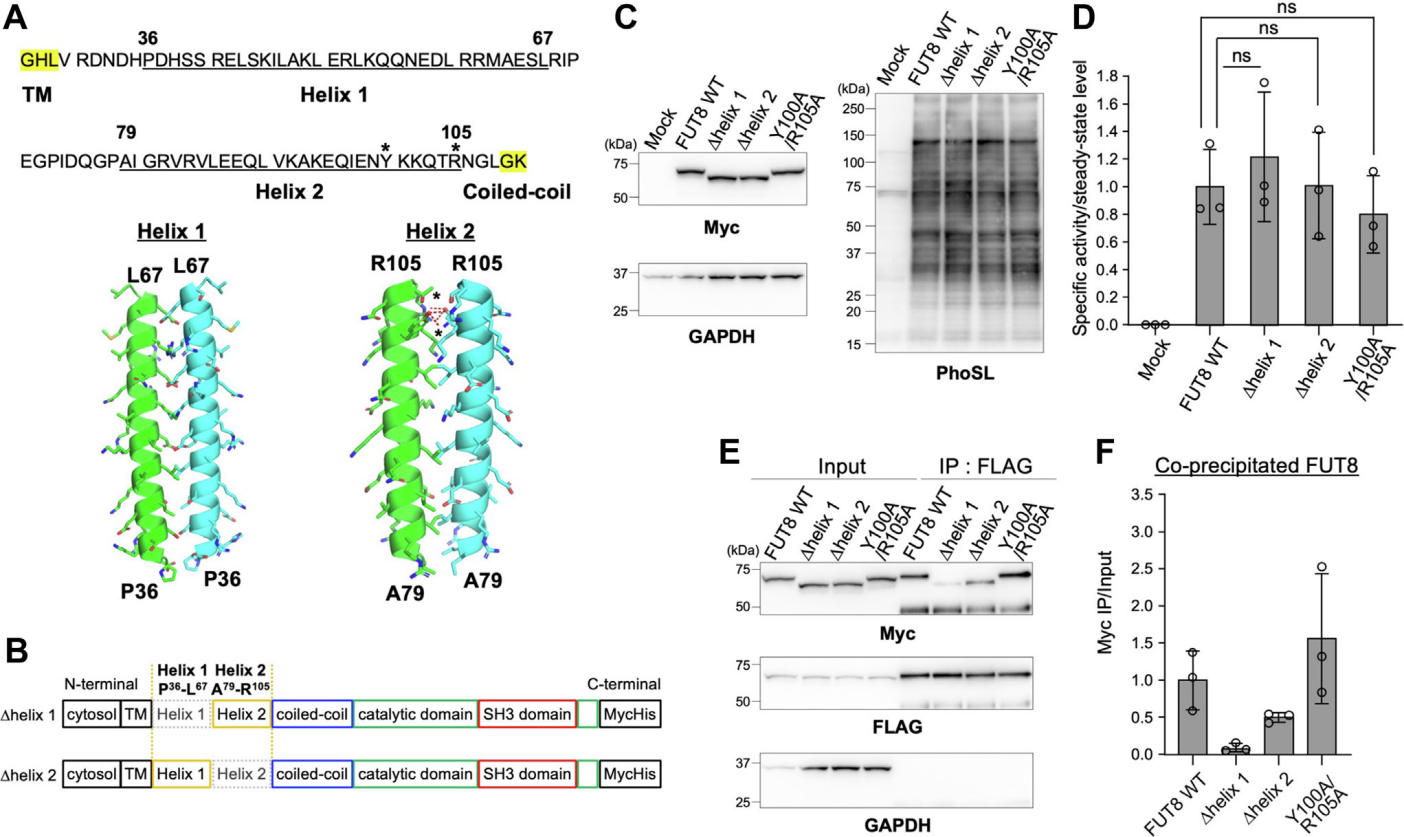
**Helix 1 in the stem region is essential for multimer formation of FUT8.***A*, amino acid sequence of FUT8 stem region and interaction models of each helix in the stem region. *Yellow* indicates residues in transmembrane or coiled-coil region. *Asterisks* indicate Tyr100 and Arg105, which might be involved in interaction. *B*, schematic structure of FUT8Δhelix 1 and Δhelix 2. *C*, HEK293 FUT8KO cells expressing FUT8 WT, FUT8Δhelix 1, Δhelix 2, or Y100A/R105A mutants were analyzed by Western blotting with anti-Myc or anti-GAPDH antibody and by lectin blotting with core fucose-specific PhoSL (expected molecular weight: FUT8Δhelix1, 68 kDa; FUT8Δhelix2, 68 kDa). *D*, lysates of HEK293 FUT8KO cells expressing FUT8 WT or mutants were incubated with the acceptor substrate and GDP-fucose, and the reaction mixture was analyzed by reverse-phase HPLC. Graph shows specific activity relative to that of FUT8 WT (*n* = 3, means ± SD, ns, not significant, Tukey’s multiple comparison test). *E*, lysates of HEK293 FUT8KO cells coexpressing Myc-FUT8 WT, Δhelix 1, Δhelix 2, or Y100A/R105A with 3xFLAG-FUT8 WT were subjected to IP with anti-FLAG antibody. Proteins in the lysates (input) or bound to the beads (IP: FLAG) were analyzed by Western blotting with anti-Myc, anti-FLAG, or anti-GAPDH antibody. *F*, quantification of Myc-FUT8 WT, Δhelix 1, Δhelix 2, or Y100A/R105 coprecipitated with 3xFLAG-FUT8. The intensity of Myc-FUT8 or its mutants coprecipitated with 3xFLAG-FUT8 was divided by that of the respective input sample in anti-Myc blot shown in (*E*). Graph shows signals relative to that of FUT8 WT (*n* = 3, means ± SD). IP, immunoprecipitation.

We next investigated whether specific regions or residues within the stem region are involved in multimer formation. The predicted secondary structure of the stem region shows that it has two helices, Helix 1 (Pro36-Leu67) and Helix 2 (Ala79-Arg105) (Figs. 3*A* and 7*A*, upper). In addition, our interaction models (Fig. 7*A*, lower), which were generated using CCBuilder 2.0 server, suggest that each helix can interact with the corresponding helix in the counterpart molecule and that Tyr100 in the Helix 2 is close enough to Arg105 in the another Helix 2 to form hydrogen bonds. Based on these predictions, we newly generated the deletion mutants FUT8Δhelix 1 and FUT8Δhelix 2 (Fig. 7*B*) and the point mutant FUT8 Y100A/R105A. Lectin blotting with PhoSL showed that the abundance of core fucosylated proteins in HEK293 FUT8KO cells expressing FUT8Δhelix 1, Δhelix 2, or Y100A/R10 A was similar to that in those expressing FUT8 WT (Fig. 7*C*). In addition, the enzymatic activities of FUT8Δhelix 1, Δhelix 2, and Y100A/R105A *in vitro* were also comparable to that of FUT8 WT (Fig. 7*D*, 1.22, 1.01 and 0.800 for FUT8Δhelix 1, Δhelix 2, and Y100A/R105A, respectively). These data again suggest that the FUT8 stem region is not essential for FUT8 activity. Next, multimer formation of FUT8Δhelix 1, Δhelix 2, and Y100A/R105A was investigated by IP-Western system. The signals for FUT8Δhelix 1 and Δhelix 2 in the anti-Myc blots were lower than that for FUT8 WT (Fig. 7, *E* and *F*, 0.0934 and 0.500 for FUT8Δhelix 1 and Δhelix 2, respectively). In particular, the abundance of coprecipitated FUT8Δhelix 1 was reduced to about 10% of that of FUT8 WT (Fig. 7*F*). FUT8 Y100A/R105A, in contrast, did not decrease FUT8 homophilic interactions (1.56) (Fig. 7*F*). These data demonstrate that both Helix 1 and Helix 2 are involved in the multimer formation of FUT8 and that Helix 1 is especially important.

### N-terminal part (cytosolic to stem region) of FUT8 is sufficient for multimer formation

**Figure 8:**
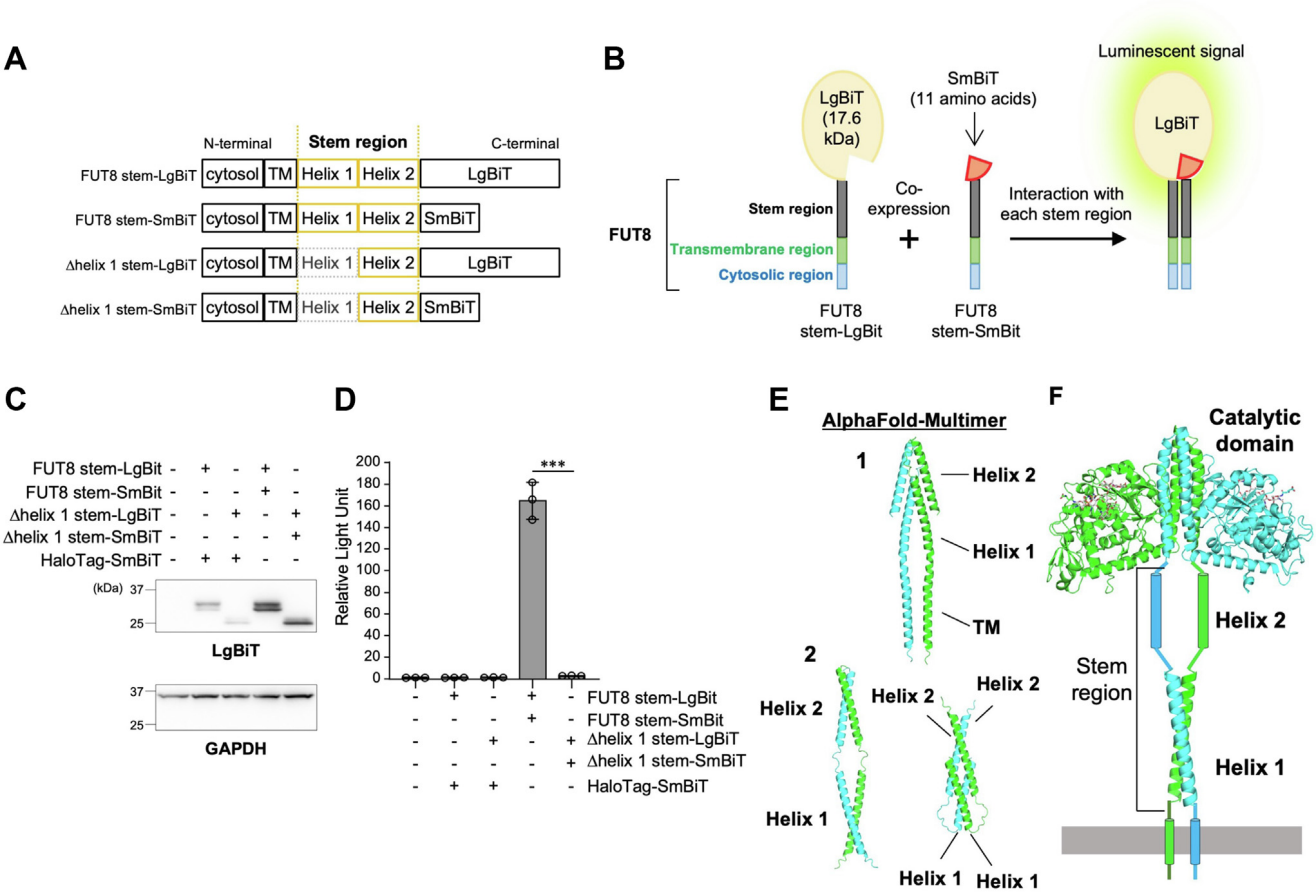
**Investigation of FUT8 multimer using NanoBiT technology and AlphaFold prediction.***A*, schematic structure of FUT8 stem-LgBiT, FUT8 stem-SmBiT, Δhelix 1 stem-LgBiT, and Δhelix 1 stem-SmBiT. *B*, overview of multimerization assay of FUT8 stem region using NanoBiT protein–protein interaction system. *C*, Western blotting analysis with anti-LgBiT or anti-GAPDH antibody. HEK293 FUT8KO cells were transfected with empty vector, pBiT1.1-C [TK/LgBiT] and pBiT2.1-C [TK/SmBiT] (Mock) (first lane), expression vectors for FUT8 stem-LgBiT and Halo-tagged SmBiT (second lane), Δhelix 1 stem-LgBiT and Halo-tagged SmBiT (third lane), FUT8 stem-LgBiT and FUT8 stem-SmBiT (fourth lane), or Δhelix 1 stem-LgBiT and Δhelix 1 stem-SmBiT (fifth lane) (expected molecular weight: FUT8 stem-LgBiT, 31 kDa; Δhelix 1 stem-LgBiT, 28 kDa). *D*, cell lysates, as used in (*C*), were incubated with NanoBiT substrate and the luminescent signal was measured. The graph shows the luminescence signal relative to that of the empty vector–transfected (Mock) sample (*n* = 3, means ± SD, ∗∗∗*p* < 0.001, Tukey’s multiple comparison test). *E*, dimer models of FUT8 stem region with transmembrane (panel 1) and without transmembrane (panel 2) regions predicted by AlphaFold-Multimer. *F*, model structure of FUT8 multimer.

As FUT8 multimerization in cells requires the FUT8 stem region, we next wondered whether the FUT8 stem region, without other luminal domains, is sufficient for multimerization. To this end, we used NanoLuc Binary Technology (NanoBiT), with which protein–protein interactions can be examined by interaction-dependent luminescence from split NanoLuc. We generated constructs coding the N-terminal part (from the cytosolic region to the stem region) of FUT8 WT and FUT8Δhelix 1 fused with a large part (LgBiT, 17.6 kDa) or small part (SmBiT, 11 amino acids) of Nanoluc (Fig. 8*A*). When the FUT8 stem regions in the constructs interacted to form multimers in cells, LgBiT and SmBiT were expected to form functional Nanoluc and emit luminescence (Fig. 8*B*). LgBiT-fused FUT8 stem or its Δhelix 1 mutant was coexpressed with its SmBiT-fused counterpart or HaloTag (negative control) in HEK293 FUT8KO cells, and expression of LgBiT-fused FUT8 stem and its Δhelix 1 mutant was confirmed by Western blotting of the cell lysates with an anti-LgBiT antibody (Fig. 8*C*). The relative luminescence of cells coexpressing FUT8 stem-LgBiT with HaloTag-SmBiT was almost the same as that of Mock cells, which were transfected with empty vectors (1.17 relative to mock) (Fig. 8*D*). However, cells coexpressing FUT8 stem-LgBiT and FUT8 stem-SmBiT showed high levels of relative luminescence (164.6), strongly suggesting that homophilic interactions involving FUT8 occurred in the cells. Moreover, the relative luminescence of cells expressing Δhelix 1 stem-LgBiT and Δhelix 1 stem-SmBiT was dramatically reduced (3.01 relative to Mock). These results indicate that the N-terminal part (from the cytosolic to stem region) of FUT8 is enough to form multimers and that Helix 1 in the FUT8 stem region is required for multimerization.

We next predicted the dimer structure of the FUT8 stem region using AlphaFold-Multimer. Prediction of the dimer structures of the FUT8 N-terminal part with a transmembrane region (Fig. 8*E*, panel 1) and without a transmembrane region (Fig. 8*E*, panel 2) showed the Helix 1-Helix 1 and Helix 2-Helix 2 interactions in all predicted forms. This suggests that both helices are involved in multimer formation of FUT8, and Helix 1, in particular, plays a critical role in multimer formation based on the biochemical results (Figs. 7*F* and 8*D*). Taken these findings into account, we generated a model for the structure of the FUT8 dimer (Fig. 8*F*).

### Reduced binding between FUT8Δstem and RPN1

**Figure 9:**
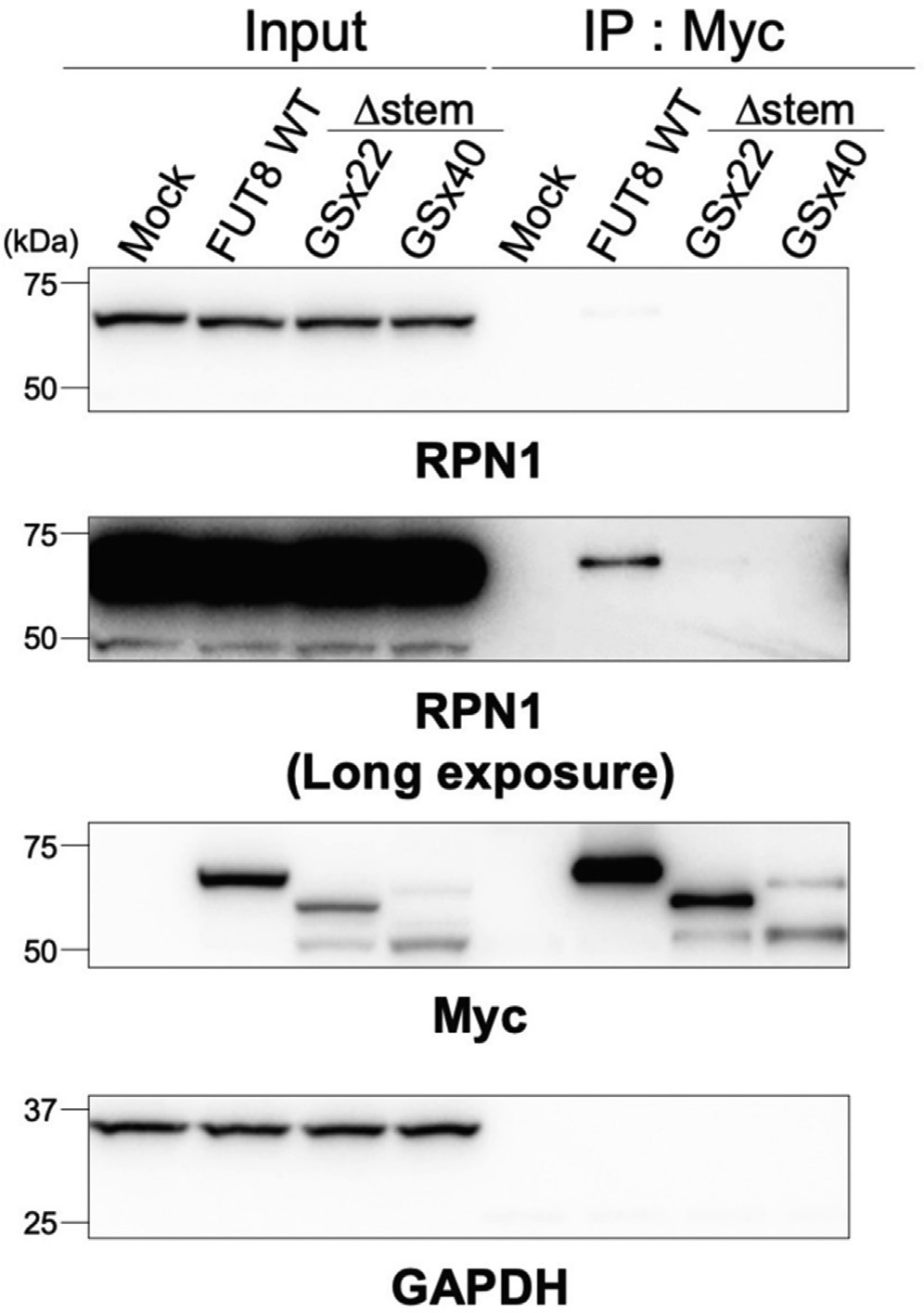
**Interaction between RPN1 and FUT8 WT, Δstem-GSx22, or Δstem-GSx40.** HEK293 FUT8KO cells were transfected with the expression plasmids encoding Myc-FUT8 WT, Δstem-GSx22, or Δstem-GSx40, and cell lysates were subjected to IP with anti-Myc antibody. Proteins in the lysates (input) or bound to the beads (IP: Myc) were analyzed by Western blotting with anti-RPN1, anti-Myc, or anti-GAPDH antibody (expected molecular weight: RPN1, 69 kDa). IP, immunoprecipitation.

Finally, we examined whether the FUT8 stem region is involved in binding with RPN1. We previously reported that RPN1, a subunit of OST, binds to FUT8 in a manner depending on the SH3 domain of FUT8 to positively regulate FUT8 activity (21). Based on these findings, the binding between Myc-tagged FUT8Δstem-GSx22 or FUT8Δstem-GSx40 and endogenous RPN1 was investigated *via* IP experiments with anti-Myc tag antibody (Fig. 9). Consistent with the previous report, RPN1 coprecipitated with FUT8 WT. In contrast, FUT8Δstem-GSx22 and FUT8Δstem-GSx40 showed almost no binding to RPN1, suggesting that the stem region of FUT8 is vital for binding to RPN1.

## Discussion

**Figure 10:**
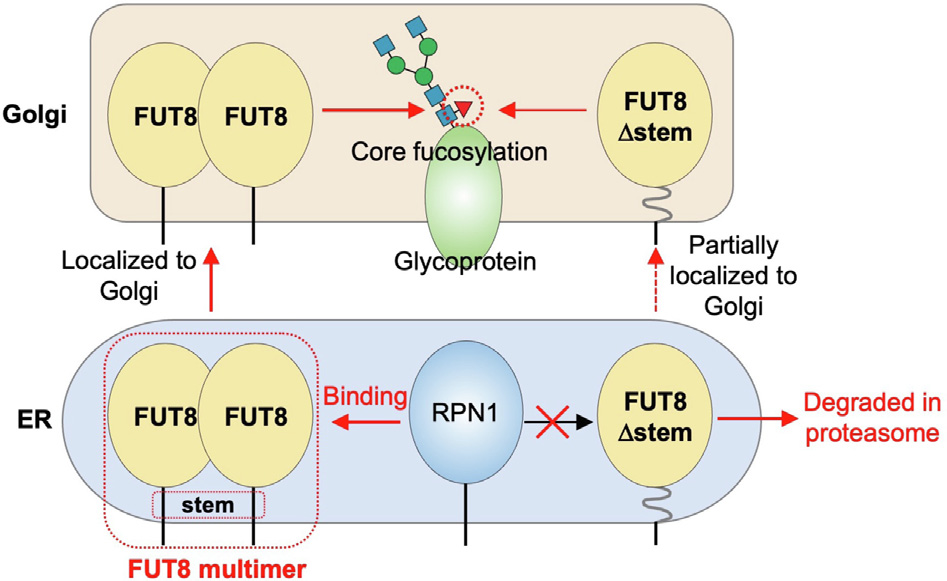
**Model of roles of the FUT8 stem region.** FUT8 WT forms a multimer through interactions between each stem region, whereas FUT8Δstem shows reduced multimer formation. The FUT8 multimer is trafficked to the Golgi apparatus and core fucosylates *N*-glycans in the Golgi. FUT8Δstem is partially localized to the Golgi but mainly degraded in the proteasome. The FUT8 stem region is also required for interaction with RPN1.

In this study, we revealed that the FUT8 stem region is essential for multimer formation, stabilization, and the RPN1 binding of FUT8 in cells (Fig. 10). As FUT8Δstem-GSx22 and FUT8Δstem-GSx40 maintained their enzyme activity and showed almost the same acceptor glycoprotein preferences as FUT8 WT (Fig. 3*B*), we concluded that the native stem region of FUT8 is not required for enzyme reactions toward glycoproteins. As there have been no reports on either the functions of the FUT8 stem region or the mechanisms of intracellular FUT8 multimer formation, our present study has provided new insights into the mechanisms of the maturation and stabilization of FUT8 in cells. Furthermore, as the multimer-less mutants FUT8Δstem-GSx22 and FUT8Δstem-GSx40 showed weak binding to RPN1, we speculated that RPN1 preferentially binds to the FUT8 multimer or that RPN1 induces FUT8 multimerization by binding to the stem region.

As to the mechanism of multimer formation *via* the stem region, it was reported that silkworm FUT8 forms a disulfide bonds between cysteines in the stem regions of monomers to form a dimer (36). The corresponding cysteine in the stem region of human FUT8 is converted to aspartic acid, and mutation of the aspartic acid to cysteine in human FUT8 was also shown to result in disulfide bonds at this position (22). Hence, the stem region of human FUT8 could be close enough to the stem region of another FUT8 monomer to interact with each other.

Our data showed that FUT8Δstem-GSx22 and FUT8Δstem-GSx40, both of which have a transmembrane region, impaired multimerization. However, recombinant soluble FUT8 fully lacking the stem region has also been reported to be a homodimer in crystal structures (29, 30), suggesting that the C-terminal region of FUT8 without the stem region also has a propensity to form a dimer. In fact, a previous study, in which the specific amino acid residues in the coiled-coil domain or SH3 domain were changed to cysteines, demonstrated that the coiled-coil domain and SH3 domain are close enough to form disulfide bonds with each other (22). In addition, size-exclusion chromatography–SAXS analysis suggested that soluble FUT8 forms a dimer in solution (30). While the coiled-coil domain and SH3 domain could contribute to the dimer formation, our data showed that multimerization of FUT8Δcoiled-coil and FUT8ΔSH3 were not impaired in the cells (Fig. 6*D*). In contrast, the multimers of FUT8Δstem-GSx22 and FUT8Δstem-GSx40 were significantly reduced despite having the intact C-terminal region (Fig. 6*C*). Furthermore, our NanoLuc assays showed that N-terminal part of FUT8 is sufficient for multimerization in the cells (Fig. 8*D*). After consideration of all these findings, we suggest that although both the stem region and the C-terminal region of FUT8 contribute to multimer formation, the stem region plays a dominant role for multimer formation in the cells.

In terms of what caused the failure in multimer formation in the cells after deleting the FUT8 stem region, there are several possibilities. First, the stem regions of FUT8 monomers could associate with each other to bring FUT8 monomers into proximity, which in turn triggers the association of catalytic domains to form the stable dimer. Because movements of the native FUT8 as a membrane protein in cells are likely to be more restricted than those of truncated soluble forms in solution, it is possible that the associations between the catalytic domains of native FUT8 require interactions between the stem regions. Second, the binding of FUT8 to RPN1 may facilitate the multimer formation of FUT8. Because our data demonstrated that FUT8Δstem-GSx22 and FUT8Δstem-GSx40 went through less multimer formation and more weakly bound to RPN1 than FUT8 WT, it is possible that RPN1 either binds to the FUT8 multimer or induces the multimer formation of FUT8 by recognizing its stem region. In native-PAGE, FUT8 WT was detected as high molecular weight complexes (Fig. 6*F*), raising a possibility that FUT8 WT interacts with several proteins, including RPN1 and other OST subunits in the cells. Although the role and mechanism of the interaction between FUT8 and RPN1 have not been fully elucidated, there are two possible roles of this interaction. A previous study reported that RPN1 acts as a chaperon for a misfolded model protein (37), and therefore, RPN1 might also bind to FUT8 to aid FUT8 folding in the ER as a chaperone. Another possibility is that RPN1 might promote core fucosylation of oligomannose type *N*-glycans by FUT8. Although *in vitro* enzymatic analysis showed that the GlcNAc residue on the α1,3-mannose arm of acceptor *N*-glycan is essential for core fucosylation (38), recent reports showed that core fucosylation also occurs toward oligomannose type *N*-glycans in the cells (39, 40), suggesting an unknown mechanism for recognition of glycoproteins with oligomannosidic glycans by FUT8. RPN1 might help FUT8 core fucosylate oligomannose glycans in the ER. Thus, a future investigation will be necessary to elucidate which FUT8 domain or region interacts with RPN1 and whether other factors are also involved in the interaction between FUT8 and RPN1.

In contrast to FUT8Δstem-GSx22 and FUT8Δstem-GSx40, FUT8Δstem-GSx2 was almost completely enzymatically inactive (Fig. 3, *B* and *C*), indicating that the stem region of FUT8 requires a certain spatial distance. In addition, while FUT8Δstem-GSx22 and FUT8Δstem-GSx40 showed similar degradation rates and multimer formation tendencies, FUT8Δstem-GSx40 also showed some differences from FUT8Δstem-GSx22, such as a lower steady-state level and more ER localization. These findings suggest that the length of the FUT8 stem region significantly impacts the folding process of the FUT8 protein. For instance, if the catalytic domain of FUT8 is not placed at an appropriate distance from the ER membrane, chaperones would not be able to properly interact with it. Such interactions between the chaperon and FUT8Δstem-GSx40 might be weaker than those with -GSx22, leading to the increased misfolding and ER entrapment of FUT8Δstem-GSx40. Additionally, the sequence of the stem region might also be important for the interaction between FUT8 and the chaperon, based on the fact that the steady-state levels of both FUT8Δstem-GSx22 and FUT8Δstem-GSx40 were lower than that of FUT8 WT, despite the sufficient lengths of the substituted GS linkers. Identification of the chaperon for FUT8 could provide insights into how the stem region of FUT8 regulates the folding process of the whole FUT8 protein.

It has been reported that some glycosyltransferases, such as ST6GAL1, are cleaved at the stem region by proteases before being secreted from the cells (41). Moreover, recent reports suggest that the amounts and activities of intracellular glycosyltransferases are regulated by their cleavage and secretion (42), indicating the importance of shedding of glycosyltransferases for glycan biosynthesis. In this study, a secreted form of FUT8 WT was detected in the culture media, and the secretion levels of FUT8Δstem-GSx22 and FUT8Δstem-GSx40 were very similar to or slightly lower than that of FUT8 WT (Fig. 4*A*). This suggests that, in the case of FUT8, the native stem region is not required for protein cleavage and secretion. As the molecular weight of extracellular FUT8 is almost the same as that of intracellular FUT8, FUT8 is likely to be cleaved intracellularly near the boundary between the transmembrane region and the stem region. Identification of the protease responsible for FUT8 secretion could unveil the significance of its secretion and presence in extracellular fluids.

Because core fucose plays important roles in the development of COPD and melanoma metastasis (4, 15), the regulation of FUT8 expression or activity is important for improving the pathology of these diseases. FUT8Δstem, which cannot form multimers, facilitated both degradation and ER localization; therefore, multimer formation *via* the stem region could be a new target for regulating core fucosylation. Furthermore, as many Golgi-localized glycosyltransferases have a stem region, the findings in this study provide important information for clarifying the general mechanisms of the subcellular localization and multimer formation of glycosyltransferases in cells. In addition to the stem region, there are cytosolic and transmembrane regions of FUT8 that have not yet undergone detailed functional analyses. In the future, dissections of the detailed functions of all domains in FUT8 will be necessary to reveal a complete picture of core fucosylation in cells, which will further lead to a fuller understanding of the biological significance of *N*-glycosylation and its medical applications.

## Experimental procedures

### Reagents

The following antibodies were used: rabbit anti-RPN1 (Abcam, ab198508), mouse anti-GAPDH (Merck Millipore, MAB374), mouse anti-Myc (Millipore, 05-724), mouse anti-FLAG (Sigma, F1804), rabbit anti-GM130 (Cell Signaling Technology, 12480), rabbit anticalnexin (Abcam, ab22595), mouse anti-APP (Millipore, MAB348), mouse anti-β-catenin (BD bioscience, 610154), mouse anti-LgBiT (Promega N7100), horseradish peroxidase (HRP)–conjugated antimouse IgG (GE Healthcare, NA931V), HRP-conjugated antisheep IgG (Abcam, ab6900), HRP-conjugated anti-rabbit IgG (GE Healthcare, NA934V), Alexa546-conjugated antisheep IgG (Invitrogen, A21098), Alexa546-conjugated antimouse IgG (Invitrogen, A10036), and Alexa488-conjugated anti-rabbit IgG (Invitrogen, A21206). Mouse anti-FUT8 (15C6) was kindly provided by Dr Eiji Miyoshi (Osaka University). Biotinylated PhoSL (26) was kindly provided by J-Oil Mils. MG132 was purchased from Merck and dissolved in dimethyl sulfoxide. CQ was purchased from Wako and was dissolved in water. CHX was purchased from Wako and was dissolved in dimethyl sulfoxide.

### Plasmid construction

Primers used for plasmid construction are listed in Table S1. FUT8 complementary DNA (cDNA) with skipping stop codon was amplified from pcDNA6.2/humanFUT8 (a kind gift from Dr Hiroaki Korekane (RIKEN)) by PCR and then ligated into *Bam*HI-*Eco*RI sites of pcDNA6-mycHisA or *Eco*RI-*Mlu*I sites of pME-3xFLAG for construction of C-terminally Myc-tagged or 3xFLAG-tagged FUT8 using NEBuilder HiFi DNA Assembly Master Mix (New England Biolabs), which was used for all ligation reactions in this study. For the construction of C-terminally Myc-tagged FUT8Δstem-GSx2, the stem region was replaced with a short glycine-serine linker (GSGS) using the methods as follows. The small fragment from the start codon to the 87th base of FUT8 cDNA and the large fragment from the 325th base to the 1725th base were amplified by PCR. These fragments were ligated into *Bam*HI-*Eco*RI sites of pcDNA6-mycHisA. For construction of the C-terminally Myc-tagged FUT8Δcoiled-coil, the coiled-coil domain was also changed to the short glycine-serine linker (GSGS), as follows. The small fragment from the start codon to 324th base of FUT8 cDNA and the large fragment from the 520th base to 1725th base were amplified by PCR and then these fragments were ligated into *Bam*HI-*Eco*RI sites of pcDNA6-mycHisA. For constructing the C-terminally Myc-tagged FUT8ΔSH3 (4Gly or 6Gly), previously constructed pcDNA6-mycHisA/FUT8ΔSH3 plasmids (4Gly or 6Gly) (21) were used as templates for PCR. The amplified fragments encoding FUT8ΔSH3 (4Gly or 6Gly) were ligated into *Bam*HI-*Eco*RI sites of pcDNA6-mycHisA. For construction of FUT8Δstem bearing the long glycine-serine linker (GSx40), a chemically synthesized GSx40 sequence (Eurofins genomics) (see Table S1 for sequence) was amplified by PCR. The PCR product and the two fragments, which were used for construction of C-terminally Myc-tagged FUT8Δstem-GSx2, were ligated into *Bam*HI-*Eco*RI sites of pcDNA6-mycHisA. The plasmid for FUT8Δstem-GSx22 was generated in the same way as that for FUT8Δstem-GSx40. The plasmids for the C-terminally 3xFLAG-tagged FUT8 deletion mutants (Δstem, Δcoiled-coil, and ΔSH3) were constructed in the same way as for pME-3xFLAG/FUT8 3xFLAG, using C-terminally Myc-tagged mutants (Δstem, Δcoiled-coil, and ΔSH3) as templates. A pcDNA-IH/FUT8 plasmids encoding soluble FUT8 (Arg68 to C terminus) was constructed as described previously (43). For the construction of C-terminally Myc-tagged FUT8 Δhelix 1, the small fragment from the start codon to the 105th base of FUT8 cDNA and the large fragment from the 202nd to 1725th base were amplified by PCR. These fragments were ligated into *Bam*HI-*Eco*RI sites of pcDNA6-mycHisA. For constructing the C-terminally Myc-tagged FUT8 Δhelix 2, the small fragment from the start codon to the 234th base and the large fragment from the 316th base to 1725th base were amplified by PCR and then these fragments were ligated into *Bam*HI-*Eco*RI sites of pcDNA6-mycHisA. A plasmid for C-terminally Myc-tagged FUT8 Y100A/R105A was constructed using the plasmid for C-terminally Myc-tagged FUT8 as a template using the Quick Change Lightning Site-Directed Mutagenesis Kit (Agilent Technologies) according to the manufacture’s protocol. To construct plasmids for FUT8 stem-LgBiT and FUT8 stem-SmBiT, a plasmid encoding Myc-tagged FUT8 was used as a template for PCR. The amplified fragment from the 1st to 326th base of FUT8 cDNA was ligated into EcoRI-XhoI sites of pBiT1.1-C [TK/LgBiT] vector (Promega) and pBiT2.1-C [TK/SmBiT] vector (Promega), respectively. To construct plasmids for Δhelix 1 stem-LgBiT and Δhelix 1 stem-SmBiT, the fragment from 1st to 230th base of FUT8 Δhelix 1 DNA sequence was amplified by PCR using Myc-tagged FUT8 Δhelix 1 as a template and then ligated into EcoRI-XhoI sites of pBiT1.1-C [TK/LgBiT] vector or pBiT2.1-C [TK/SmBiT] vector.

### Cell culture

HEK293 (purchased from ATCC), HEK293/FUT8KO, COS7 (purchased from RIKEN cell bank), and HeLa (purchased from RIKEN cell bank) cells were grown at 37 °C under 5% CO_2_ conditions in Dulbecco's modified Eagle's medium (DMEM) supplemented with 10% fetal bovine serum and 50 μg/ml kanamycin. Dr Jianguo Gu (Tohoku Medical and Pharmaceutical University) kindly provided the HEK293/FUT8KO cells, which were generated by Zn-Finger technology. To investigate FUT8 degradation, MG132 (10 μM final concentration) or CQ (50 μM final concentration) was added to the culture medium 48 h after transfection. After 48 h, the cells were collected for Western blotting.

### Plasmid transfection

Transient transfection was performed on approximately 5.0 × 10^6^ or 2.0 × 10^6^ cells grown on a 10-cm or 6-cm dish using Lipofectamine 3000 transfection reagent (Thermo Fischer Scientific) following the manufacture’s protocol. Cells on a 10-cm or 6-cm dish were transiently transfected with 10 μg or 4 μg of each plasmid. For expression of recombinant soluble FUT8, 30 μg of the plasmid was transfected into COS7 cells plated on a 15-cm dish using PEI MAX (Polyscience).

### Western and lectin blotting and CBB staining

Cells were collected and sonicated in lysis buffer (Tris-buffered saline [TBS] containing 1% Triton X-100 and protease inhibitor mixture [Roche Applied Science]). After centrifugation of the cell lysates, the supernatants were collected, and protein concentrations of the supernatants were measured using a Pierce bicinchoninic acid (BCA) Protein Assay (Thermo Fisher Scientific) according to the manufacturer’s protocol. The cell lysates were mixed with Laemmli SDS sample buffer and then subjected to 5% to 20% SDS-PAGE and Western and lectin blotting. For CBB staining, after SDS-PAGE, the gel was incubated with GelCode Blue Safe Protein Stain (Thermo Fisher Scientific) and imaged using FUSION-SOLO 7s EDGE (Vilber-Lourmat). For Western blotting, proteins separated by SDS-PAGE were transferred to a nitrocellulose membrane. After blocking the membranes with TBS containing 5% skim milk and 0.1% Tween-20, they were incubated with the primary antibodies overnight at 4 °C. After washing with TBS containing 0.1% Tween-20, the membranes were incubated with the HRP-conjugated secondary antibodies at room temperature (RT). After washing with TBS, signals were detected with Western Lightning Plus-ECL (PerkinElmer) or SuperSignal West Femto Maximum Sensitivity substrate (Thermo Fisher Scientific) using FUSION-SOLO 7s EDGE (Vilber-Lourmat). Signals showing protein bands were quantified using FUSION-SOLO 7s EDGE (Vilber-Lourmat). For lectin blotting, after protein transfer, membranes were blocked with TBS containing 0.1% Tween-20. After blocking, the membranes were incubated with the biotinylated lectin overnight at 4 °C, washed with TBS containing 0.1% Tween-20, followed by incubation with HRP-streptavidin (VECTASTAIN ABC Standard Kit). After washing with TBS, the protein bands were detected in the same way as for the Western blot. The data in the figures are representative of three or more experiments.

## Native PAGE

Sample preparation for Blue Native PAGE was performed using NativePAGE Sample Prep Kit (Novex) according to the manufacturer’s protocol. Briefly, cells were suspended on ice in sample buffer (supplied in the kit) containing 1% digitonin and protease inhibitor mixture (Roche Applied Science). After ultracentrifugation at 100,000*g* for 15 min at 4 °C, the supernatants were collected, and protein concentrations were measured using Pierce BCA Protein Assay (Thermo Fisher Scientific), followed by adding the NativePAGE G-250 (final concentration was 1/4 of detergent concentration). Proteins were separated by 4% to 16% native PAGE with NativePAGE running buffer (Novex) and cathode buffer (Novex) and then transferred to a polyvinylidene difluoride membrane. Proteins on the polyvinylidene difluoride membrane were fixed with 8% acetic acid, and the membranes were washed with methanol. The methods for blocking, antibody incubation, and signal detection were the same as those described previously for Western blotting.

### Purification of recombinant FUT8

The purification of recombinant soluble FUT8 was carried out as described previously (43). Briefly, 60% to 70% confluent COS7 cells on 15-cm dishes were transfected with the expression plasmids using PEI MAX. After 6 h of transfection, the culture media were removed, and the cells were incubated with Opti-MEM I for 72 h at 37 °C. The culture media were collected, and the cell debris was removed by centrifugation. Then, 6xHis-tagged FUT8 was purified from the medium using a Ni^2+^ column and then desalted using a NAP-5 gel filtration column (GE Healthcare).

### FUT8 activity assay

The assay for FUT8 activity was performed as described previously (27, 28). Briefly, 0.3 μg of protein in a cell lysate expressing FUT8 was incubated in 10 μl of reaction buffer (100 mM Mes-NaOH, pH 7.0, 200 mM GlcNAc, 0.5% Triton X-100, 1 mg/ml bovine serum albumin) containing 1 mM GDP-fucose and 10 μM fluorescently labeled acceptor substrate (GnGn-bi-Asn-PNSNB [N-(2-(2-pyridylamino) ethyl) succinamic acid 5-norbornene-2,3-dicarboxyimide ester]) at 37 °C for 20 min. The reaction mixture was boiled at 95 °C for 5 min to stop the reaction, followed by the addition of 40 μl of water. The mixture was centrifugated at 21,500*g* for 5 min, and 10 μl of the supernatant was injected to an HPLC machine equipped with an ODS column (Tosoh, 4.6 × 150 mm) to detect the fluorescence-conjugated acceptor substrate and product. The mobile phase consisted of 80% buffer A (20 mM ammonium acetate) and 20% buffer B (buffer A including 1% 1-butanol).

### Immunofluorescence staining

Cells on an 8-well glass chamber slide were fixed with 4% PFA/PBS at RT for 15 min, washed with PBS for three times, followed by permeabilization with 0.1% Triton X-100/0.1% bovine serum albumin/PBS at RT for 15 min. After washing the cells with PBS three times, they were incubated with primary antibodies for 60 min and then washed with PBS. Next, Alexa488- or Alexa546-conjugated secondary antibodies and 4′,6-diamidino-2-phenylindole were added to the cells, followed by further incubation for 30 min. After washing with PBS, the chamber was removed, and the mounting fluid (ProLong Diamond Antifade Mountant, Invitrogen) was used to cover the cells, followed by a cover glass. Fluorescence was imaged using a BZ-X800 all-in-one fluorescence microscope (KEYENCE). Quantification of intensities was carried out using ImageJ Fiji software (https://fiji.sc): we first converted the acquired images to 8 bit and outlined the calnexin or GM130 signals, then the R value of Pearson correlation coefficient between the calnexin or GM130 and the Myc signal in the enclosed parts were calculated by Coloc 2.

### IP

Cells were suspended in lysis buffer (TBS containing 1% Triton X-100 and protease inhibitor mixture) and lysed with sonication. The cell lysates were ultracentrifuged at 100,000*g* for 20 min, and the supernatants were collected. An aliquot of the collected supernatant was used as input for Western blotting and the rest was incubated with Anti-DYKDDDDK tag antibody magnetic beads (FUJIFILM) at 4 °C overnight. The beads were washed three times with 0.1% Triton X-100/PBS and then boiled with 2× Laemmli SDS sample buffer at 95 °C for 5 min to elute the proteins bound to the beads. For immunoprecipitation with the anti-Myc antibody, the rest of the supernatant after ultracentrifugation was prepared as aforementioned and precleared with Dynabeads protein G (Thermo Fisher Scientific) at 4 °C for 30 min. The precleared lysates were incubated with anti-Myc antibody and Dynabeads protein G at 4 °C overnight and then washed three times with 0.1% Triton X-100/PBS. The beads were boiled with 2 × Laemmli SDS sample buffer at 95 °C for 5 min. The samples were analyzed by SDS-PAGE and Western blotting.

### CHX chase

HEK293 FUT8KO cells on 6-cm dishes were transfected with the expression plasmids. After 24 h, the culture media were removed, and the cells were incubated with 100 μg/ml CHX/10% fetal bovine serum/DMEM at 37 °C. Then, 0, 4, 8, or 24 h after adding CHX, cells were collected and used for Western blotting.

### Protein preparation from cell culture medium

HEK293 FUT8KO cells were transfected with the expression plasmids. After 6 h, the culture media were removed, and the cells were incubated with Opti-MEM I at 37 °C for 48 h. The cells and the culture media were collected, and the collected cells lysed for SDS-PAGE. The collected culture media were ultracentrifuged at 100,000*g* at 4 °C for 20 min and the supernatants were collected. A 1/30 volume of 5 M NaCl and 1/2.5 volume of EtOH were added, followed by incubation at −80 °C for 10 min. After centrifugation at 12,000*g* at 4 °C for 30 min, the precipitates were washed with 70% EtOH and centrifugated at 16,200*g* at 4 °C for 5 min. The precipitates were then suspended in Laemmli SDS sample buffer. After boiling at 95 °C for 5 min, the samples were used for SDS-PAGE and Western blotting.

### Prediction of FUT8 secondary structure

The amino acid sequence of human FUT8 was retrieved from the UniProtKB database (accession code: Q9BYC5). The obtained sequence was subjected to secondary structure prediction on the server PSIPRED 4.0 (http://bioinf.cs.ucl.ac.uk/psipred/).

### NanoBiT protein–protein interaction assay

Protein–protein interactions were measured using NanoBiT Protein:Protein Interaction System (Promega, N2014). HEK293 FUT8KO cells cultured on a 6-cm dish were transfected with empty vector or expression vector, collected, and sonicated in lysis buffer (TBS containing 1% Triton X-100), 48 h after transfection. Cell lysates were centrifuged at 21,500*g* at 4 °C for 20 min and the supernatant was collected. Protein concentrations of the supernatant were measured using Pierce BCA Protein Assay. In a 96-well plate, 0.6 μg of proteins in the cell lysate were diluted in 100 μl of TBS in a well, and the diluted lysate was incubated at RT with 25 μl of Nano-Glo Live Cell Substrate that had been diluted with Nano-Glo LCS dilution buffer according to the manufacturer’s protocol. Luminescence signal was measured using Synergy H1 (BioTek).

### Structural representation

The dimerization models of two putative α-helices (P36−L67, A79−R105) in Figure 7*A* were generated by CCBuilder 2.0 server (http://coiledcoils.chm.bris.ac.uk/ccbuilder2/builder). The dimer models of the FUT8 stem region in Figure 8*E* were generated by the AlphaFold-Multimer program (44). The model structure of FUT8 dimer in Figure 8*F* was generated using the reported dimer structure of FUT8 catalytic domain (Protein Data Bank code: 6VLD) and the dimerization model of Helix 1 in Figure 7*A*. All structural figures were prepared with PyMOL (The PyMOL Molecular Graphics System, Version 1.2r3pre, Schrödinger, LLC.).

### Statistical analysis

Statistical analysis was performed using GraphPad Prism 8 software (GraphPad Software, Inc).

## Data availability

All the data are contained within the article.

## Supporting information

This article contains supporting information.

## Conflict of interest

The authors declare that they have no conflicts of interest with the contents of this article.
