# Supplementary material for: The stem region of α1,6-fucosyltransferase FUT8 is required for multimer formation but not catalytic activity
Source: J Biol Chem. 2022 Nov 3;298(12):102676. doi: 10.1016/j.jbc.2022.102676 (PMC9709245; doi:10.1016/j.jbc.2022.102676)
Supplement: Supplementary information [file mmc1.pdf]

Supplementary Information

The stem region regulates multimer formation of α1,6-fucosyltransferase FUT8

Seita Tomida, Masamichi Nagae, Yasuhiko Kizuka*

Correspondence: Yasuhiko Kizuka, Ph.D.
Email: kizuka@.gifu-u.ac.jp

This Supplemental information includes:

Table S1

Table S1

Primer_Name	Sequence
FUT8 mycHis Fw	TTAAGCTTGGTACCGAGCTCGGATCCGCCACCATGCGGCCATGGAC
FUT8 mycHis Rv	GCTGGATATCTGCAGAATTCTTTCTCAGCCTCAGGATATG
FUT8 3xFLAG Fw	CTTCTGCTCTAAAAGCTGCGGAATTCGCCACCATGCGGCCATGGAC
FUT8 3xFLAG Rv	TCATCCTTGTAGTCTCCGCCACGCGTTTTCTCAGCCTCAGGATATG
FUT8_Δstem-GSx2_Fw	GTCACTTGGGATCAGGCTCTGGGAAGGATCATGAAATCCT
FUT8_Δstem-GSx2_Rv	TCCTTCCCAGAGCCTGATCCCAAGTGACCACCTATATAAA
FUT8_Δ-coiled-coil_Fw	ATGGTCTGGGATCAGGCTCTGATGGAGCAGGTGATTGGCG
FUT8_Δcoiled-coil_Rv	GCTCCATCAGAGCCTGATCCCAGACCATTTCTGGTCTGTT
delta stem GSx40_Fw	TTTATATAGGTGGTCACTTG
delta stem GSx40_Rv	AGGATTTCATGATCCTTCCC
FUT8 Δ helix1_Fw	TGGTACGAGATAATGACCATCGGATACCAGAAGGCCCTAT
FUT8 Δ helix1_Rv	ATAGGGCCTTCTGGTATCCGATGGTCATTATCTCGTACCA
FUT8 Δ helix2_Fw	GCCCTATTGATCAGGGGCCAAATGGTCTGGGGAAGGATCA
FUT8 Δ helix2_Rv	TGATCCTTCCCCAGACCATTTGGCCCCTGATCAATAGGGC
FUT8 Y100A/R105A_Fw	GAACAGATTGAAAATGCCAAGAAACAGACCGCAAATGGTCTGGGG
FUT8 Y100A/R105A_Rv	CCCCAGACCATTTGCGGTCTGTTTCTTGGCATTTTCAATCTGTTC
FUT8 stem NanoBit_Fw	GCCTAAGTGGGAGCTCAGGGGAATTCGCCACCATGCGGCCATGGAC
FUT8 stem NanoBit_Rv	CTCCGCTCCCGCCACCACCGCTCGAGCCCAGACCATTTCTGGTCTG

Name	Sequence
80x Glycine/Serine Linker	TTTATATAGGTGGTCACTTGGGATCAGGTAGCGGCTCAGGGAGCGGA<br>TCGGGTTCGGGTAGCGGTTCCGGGTCCGGGTCGGGTTCGGGGTCTGGT<br>AGTGGCTCGGGCAGCGGCTCGGGATCTGGCAGCGGATCAGGATCGG<br>GATCAGGCTCAGGGTCTGGCTCCGGCTCTGGCAGTGGTTCCGGGTCGG<br>GATCGGGGTCGGGGTCAGGTTCCGGCAGCGGTTCCGGATCTGGTAGT<br>GGGAGTGGTAGCGGGTCCGGATCGGGGAAGGATCATGAAATCCT
